# Protective effects of mesenchymal stromal cell-derived secretome on dermonecrosis induced in rabbits by *Loxosceles intermedia* spider venom

**DOI:** 10.1590/1678-9199-JVATITD-2024-0004

**Published:** 2024-07-22

**Authors:** Gabriela Marques Rodrigues, Mara Elvira de Almeida, Sóstenes Apolo Correia Marcelino, Paula Bretas Ullmann Fernandes, Jessica Oliveira Pereira da Cruz, Françoise Louanne Araújo, Raquel da Silva Ferreira, Ana Flávia Machado Botelho, Francisco Javier Bedoya, Gladys Margot Cahuana, Ana Belén Hitos, Bernat Soria, Fernanda Costal-Oliveira, Clara Guerra Duarte, Juan R. Tejedo, Carlos Chávez-Olórtegui, Marília Martins Melo

**Affiliations:** 1 Department of Veterinary Clinic and Surgery, Veterinary College, Federal University of Minas Gerais (UFMG), Belo Horizonte, MG, Brazil.; 2 Department of Molecular Biology and Biochemical Engineering, Universidad Pablo de Olavide, Seville, Spain.; 3 Biomedical Research Network for Diabetes and Related Metabolic Diseases (CIBERDEM), Instituto de Salud Carlos III, Madrid, Spain.; 4 Institute of Bioengineering and Institute of Biomedical Research ISABIAL, University Miguel Hernández de Elche, Alicante, Spain.; 5 Department of Biochemistry and Immunology, Institute of Biological Sciences, Federal University of Minas Gerais (UFMG), Belo Horizonte, MG, Brazil.; 6 Ezequiel Dias Foundation, Belo Horizonte, MG, Brazil.; 7 Institute of Tropical Diseases, Universidad Nacional Toribio Rodríguez de Mendoza de Amazonas, Chachapoyas, Peru.

**Keywords:** Loxoscelism, Loxosceles intermedia, Regenerative therapy, Secretome

## Abstract

**Background:**

Loxoscelism refers to a set of clinical manifestations caused by the bite of spiders from the *Loxosceles* genus. The classic clinical symptoms are characterized by an intense inflammatory reaction at the bite site followed by local necrosis and can be classified as cutaneous loxoscelism. This cutaneous form presents difficult healing, and the proposed treatments are not specific or effective. This study aimed to evaluate the protective effect of mesenchymal stromal cells-derived secretome on dermonecrosis induced by *Loxosceles intermedia* spider venom in rabbits.

**Methods:**

Sixteen rabbits were distributed into four groups (n = 4). Except for group 1 (G1), which received only PBS, the other three groups (G2, G3, and G4) were initially challenged with 10 μg of *L. intermedia* venom, diluted in 100 μL of NaCl 0.9%, by intradermic injection in the interscapular region. Thirty minutes after the challenge all groups were treated with secretome, except for group 2. Group 1 (G1-control group) received intradermal injection (ID) of 60 μg of secretome in 0.15 M PBS; Group 2 (G2) received 0.9% NaCl via ID; Group 3 (G3) received 60 μg of secretome, via ID and Group 4 (G4), received 60 μg of secretome by intravenous route. Rabbits were evaluated daily and after 15 days were euthanized, necropsied and skin samples around the necrotic lesions were collected for histological analysis.

**Results:**

Rabbits of G1 did not present edema, erythema, hemorrhagic halo, or necrosis. In animals from G2, G3, and G4, edema appeared after 6h. However, minor edema was observed in the animals of G2 and G3. Hemorrhagic halo was observed in animals, six hours and three days after, on G2, G3, and G4. Macroscopically, in G4, only one animal out of four had a lesion that evolved into a dermonecrotic wound. No changes were observed in the skin of the animals of G1, by microscopic evaluation. All animals challenged with *L. intermedia* venom showed similar alterations, such as necrosis and heterophilic infiltration. However, animals from G4 showed fibroblast activation, early development of connective tissue, neovascularization, and tissue re-epithelialization, indicating a more prominent healing process.

**Conclusion:**

These results suggest that secretome from mesenchymal stromal cells cultured in a xeno-free and human component-free culture media can be promising to treat dermonecrosis caused after *Loxosceles* spiders bite envenoming.

## 1. Background

Loxoscelism refers to a set of clinical manifestations caused by the bite of spiders from the *Loxosceles* genus. Two different conditions triggered by this envenoming are the most common: (a) the cutaneous form, which causes local reactions and a dermonecrotic wound with gravitational propagation, that is difficult to heal; (b) the visceral-cutaneous form, which leads to manifestations of renal failure and hematological disorders [1, 2]. 

Although antivenoms effectively reverse some effects induced by *Loxosceles* envenoming [3], they have a limited therapeutic role, unable to completely neutralize the venom’s local effects. Despite its existence in human accident treatment, many people develop disfiguring scars, necrotic crusts, and skin infections, which can lead to permanent sequelae with physical, social, and psychological implications [4]. In human medicine various interventions have been proposed: dapsone, surgical excision, steroids, hyperbaric oxygen, and antivenom therapy [5, 6]. Unlike human medicine, in veterinary medicine, anti-loxoscelic antivenom for specific treatment is not available. In animals’ cases of loxoscelism, treatment is instituted according to observed clinical signs, including broad-spectrum antibiotics, corticosteroids, antihistamines, and surgical wound excision [7, 8]. Therefore, it is necessary to search for alternatives to improve the expectations of *Loxosceles* envenoming treatment, both in humans and animals, being the mesenchymal stem/stromal cells (MSCs)-based therapy a promising possibility. 

MSCs are derived from different tissues, which present regenerative [9], immunomodulatory [10], antitumoral [11, 12], and antimicrobial properties [13]. Presently, MSCs are primarily involved in facilitating skin wound healing through the paracrine function of multiple factors. Of note, the therapeutic utilization of MSCs in wound healing is also limited by storage challenges, mutation-related tumorigenicity, optimal cell activity, immune rejection, and ethical factors [14]. Studies have revealed that implanted cells do not survive for long [15-17]. It has been reported that < 1% of MSCs survive for more than one week after systemic administration [18, 19] and that, the benefits of MSC therapy could be due to the vast array of bioactive factors they produce, which play an important role in the regulation of key biologic processes [20]. 

Cell-based therapy involves the direct use of cells or the use of exocrine products derived from their culture, known as secretome or MSCs-derived exosomes [21], obtained from secretome ultracentrifugation. Therefore, the secretome from MSCs has attracted much attention for its potential use in tissue repair and regeneration [22, 23]. We have recently published a review on MSCs and muscle regeneration in the context of snakebite envenoming, in which we proposed that MSCs based therapies can induce muscle regeneration, mainly by anti-inflammatory activity, paracrine effects, revascularization induction, and microenvironment remodeling [9]. Adipose-derived MSCs are also very commonly utilized in wound healing applications due to its high accessibility, minimal invasiveness, and lack of ethical limitations [24]. Recently, exosomes that were derived from adipose-derived MSCs have shown accelerated wound healing with a significant increase in the wound closure rate by attenuating the inflammation phase [25, 26]. Preliminary results of this study suggest that secretome reduces acute muscle damage produced by *B. atrox* venom [9]. In addition, exosomes derived from both adipose tissue and bone marrow have been reported to be able to revert macrophage activation, downregulation of cytokine storm, and hyperinflammatory state associated with COVID-19, showing that MSCs block tissue damage and promote recovery, which can extend their application to a wide range of inflammatory diseases [27, 28]. This is a promising scenario, as the use of MSCs-based therapies may be beneficial for healing the tissue damage caused by the *Loxosceles* spider bite. 

Delays in patients seeking medical care may contribute to the extent of local tissue damage because the skin necrosis induced by *Loxosceles* venom begins within hours of envenoming [2]. Therefore, the use of a treatment capable of inhibiting the formation of necrosis or promoting better or faster wound healing, certainly, will contribute to the restoration of the clinical condition. It is important to highlight that there are no studies that use the secretome as a treatment for dermonecrotic wounds caused by loxoscelism, therefore, this research is original.

Given this context, the present work aims to evaluate the potential of MSC’s secretome in preventing and healing dermonecrotic lesions experimentally caused by *Loxosceles intermedia* spider venom in rabbits. 

## 2. Methods

### 2.1. Animals

This study was conducted in accordance with ethical principles, respecting animal welfare, and was approved by the Ethics Committee for the Use of Animals (CEUA) of the Federal University of Minas Gerais, CEUA protocol N^o^ 131/2020.

Male New Zealand rabbits (weighing approximately 2.0 kg), were purchased from the Experimental Farm *Professor Hélio Barbosa*, from the Veterinary School of the Federal University of Minas Gerais (EV-UFMG) Brazil. Animals were maintained in individual metallic cages (30 × 30 × 75 cm) at the Metabolism and Calorimetry Lab of EV-UFMG, receiving water and food *ad libitum.*

### 2.2. Venoms and MSC’s secretome

A venom pool, extracted by electrostimulation from adult male and female *Loxosceles intermedia* spiders, was provided by *Centro de Produção e Pesquisa em Imunobiológicos* (CPPI), Paraná, Brazil. The secretome was provided by Dr. Juan R. Tejedo from Molecular Biology and Biochemical Engineering of University of Pablo de Olavide, Seville, Spain, as a lyophilized product originated from allogenic Mesenchymal Stromal Cells derived from human adipose tissue (adMSC) cultivated in a xeno-free and human component-free culture media, named XANADU media modified from [29] and was used as a suspension with protein concentration of 0.57 mg/mL.

### 2.3. Experimental design

Animals were adapted to captivity for seven days and were then separated into four groups of four animals (n = 4) each. Except for group 1 (G1), which received only PBS, the other three groups (G2, G3, and G4) were initially challenged with 10 μg of *L. intermedia* venom, diluted in 100 μL of NaCl 0.9%, by intradermic injection in the interscapular region. Thirty minutes later, animals were treated as described in Table 1. Animals from group 2 received only saline, intradermally. Rabbits from groups 1, 3, and 4 received 60 μg of secretome in PBS [9]. Groups 1 and 3 received the secretome in four equidistant points (15 μg per point), in the interscapular region by intradermal route, and group 4, received the secretome by the intravenous route, in the lateral marginal atrial ear vein.

**Table 1.** Distribution of rabbits in different groups and treatment protocols after injection of *Loxosceles intermedia* venom.

GROUP	Challenge (i.d via)	Treatment
G1	PBS	Secretome (60 μg) in PBS - i.d.
G2	*L. intermedia* venom (10 μg) in PBS	0.9% NaCl - i.d.
G3	*L. intermedia* venom (10 μg) in PBS	Secretome (60 μg) in PBS - i.d.
G4	*L. intermedia* venom (10 μg) in PBS	Secretome (60 μg) in PBS (i.v., lateral marginal auricular vein)

PBS: phosphate-buffered saline; NaCl: sodium chloride solution - saline solution); i.d.: intradermic; i.v.: intravenous.

To monitor lesion evolution and treatment performance, for each group, the lesions were measured daily and dermatological aspects such as erythema, edema, hemorrhagic halo, and necrosis were evaluated. Daily photographic records were taken with a digital camera and kept at a constant distance of 30 cm from the lesion. The lesions were evaluated, and measured on the first day (six hours after treatment), third, ninth, and fifteenth days. 

### 2.4. Histomorphologic evaluation

After a period of 15 days of treatment and observation, animals were euthanized by deepening anesthesia with intravenous propofol (> 10 mg/kg) and potassium chloride (1.0 mL/kg). Skin samples were collected, fixed in 10% formalin, and processed by a routine technique of embedding in paraffin to perform 4 µm-thick histological sections. Slices were stained using the Hematoxylin-Eosin (HE) technique for histomorphometry evaluation using conventional light microscopy analysis. A descriptive analysis of all slides was performed, blinded for the animal group. Changes in the morphological structure of the epidermis, superficial and deep dermis, and muscle layer were evaluated.

### 2.5. Statistical analysis

The experimental design was completely randomized and data were presented as mean ± standard deviation. The normality test of Kolmogorov-Smirnov was used to analyze quantitative variables, followed by an analysis of variance (ANOVA) and the Tukey test. The non-normal data were analyzed using the Kruskal-Wallis non-parametric test, Dunn’s multiple comparisons, and the Friedman test for matched samples. The significance level established was *p < 0.05*. Data were evaluated by GraphPad Prism v.8.0.

### 3. Results

### 3.1. Macroscopic evaluation of dermonecrosis

**Figure 1.**
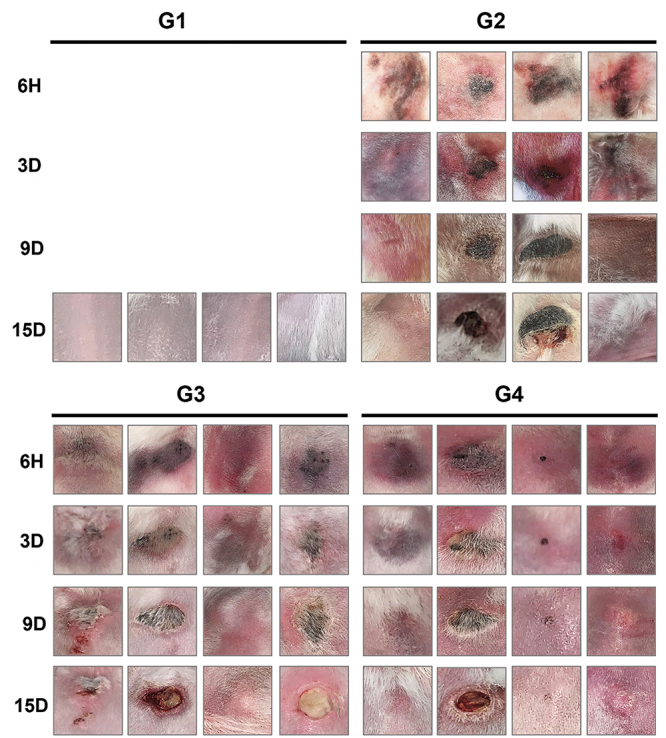
Evolution of rabbit dermonecrotic wound after challenged with *Loxosceles intermedia* venom and treatment with 0.9% NaCl (G2); after injection of *Loxosceles intermedia* venom and treatment with ID secretome (G3); and after injection of *L. intermedia* venom and treatment with IV secretome (G4). Group 1 (G1) was challenged with PBS and treated with secretome, and no macroscopic changes were observed.

Lesions started to develop in all the animals that received *L. intermedia* venom, with or without secretome treatments. However, not all animals presented the classic dermonecrotic wound. In most animals, the lesion evolved with gravitational spreading, being accompanied by dermal necrosis 72 hours after venom injection. After this time, it was observed that a central area of necrosis evolved from the hemorrhagic halo in animals from (G2), which received venom and only saline as treatment. Subsequently, nine days after venom injection, a crust was formed in the necrotic area, that detached almost completely on the 15th day (Figure 1). 

G1 animals that were challenged with PBS and treated with the secretome, did not develop injuries, such as erythema, hemorrhagic halo, or necrosis. Only a very mild edema was detected in G1, suggesting that secretome alone does not cause local adverse effects.

**Figure 2.**
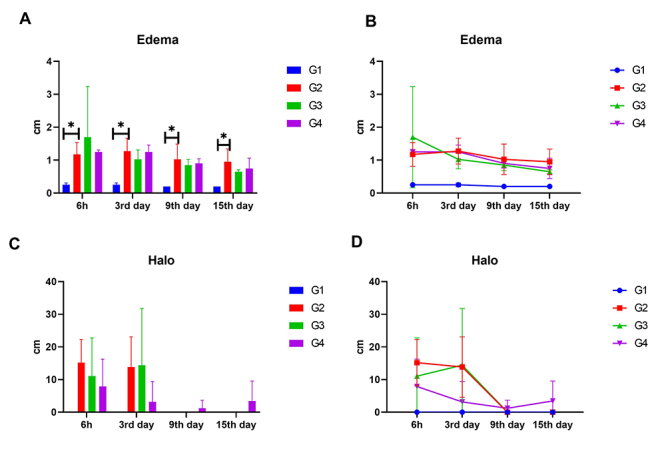
(A) Edema mean values (cm) of rabbits challenged with PBS and treated with secretome (G1) and challenged with *L. intermedia* venom and treated with 0.9% NaCl (G2), with secretome intradermally (G3) and intravenous (G4), at time 1 (six hours), third, ninth, and fifteenth day after challenge and treatments. (B) Global mean value ± standard deviation of edema of rabbits challenged with PBS and treated with secretome (G1) and challenged with *L. intermedia* venom and treated with 0.9% NaCl (G2), with secretome intradermally (G3) and intravenous (G4), after challenge and treatments. (C) Hemorrhagic halo values (cm) of rabbits challenged with PBS and treated with secretome (G1) and challenged with *L. intermedia* venom and treated with 0.9% NaCl (G2), with secretome intradermally (G3) and intravenous (G4), at time 1 (six hours), third, ninth, and fifteenth day after challenge and treatments. (D) Global mean value ± standard deviation of hemorrhagic halo of rabbits challenged with PBS and treated with secretome (G1) and challenged with *L. intermedia* venom and treated with 0.9% NaCl (G2), with secretome intradermally (G3) and intravenous (G4), after challenge and treatments.

All animals that received the venom presented edema and hemorrhagic halo six hours and three days after application, with consequent reduction after 9 and 15 days (Figure 2). 

A hemorrhagic halo was not observed in G1 but was present in the groups that received venom (G2, G3, and G4). At 6 hours and the third day, bigger halos were observed in G2, but the values were reduced in the following times. Interestingly, on the ninth day, G4 still had a halo, associated with a single animal, in which the lesion was still present. It is important to point out that, macroscopically, only one animal evolved to a dermonecrotic wound in this group. However, this was not statistically significant (Figures 2 C  and 2D). The only standard deviation superior to 15 was presented by group G3 on the third day. This can be traced to the individual values of the group: animal 1 (1.76), animal 2 (38.46), animal 3 (15.89) and animal 4 (1.53). Two animals of the group had a discrete halo, whilst the other two had significant lesions, especially animal number 2, justifying the high values of standard deviation (SD).

### 3.2. Microscopic evaluation of dermonecrosis

**Figure 3.**
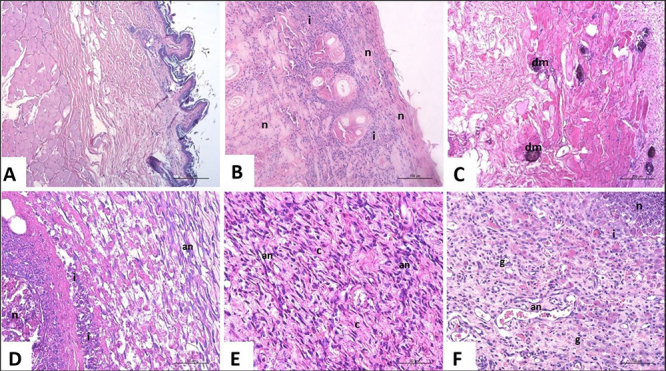
Histological image of skin from rabbits inoculated with *L. intermedia* venom after HE staining. **(A)** Rabbit of G1. Normal skin is seen, with no histological changes in the skin layers. **(B)** Rabbit of G2. Note that in the epidermis, superficial, deep dermis, extending to the hypodermis, there are multifocal to coalescent areas of necrosis (n), and tissue loss with accentuated heterophilic inflammatory infiltrate (i). **(C)** Rabbit of G2. Multifocal areas of vitreous basophilic material deposition are observed, with evidence of dystrophic mineralization (dm) in the final assessment. **(D)** Rabbit of G3. In greater magnification, intense heterophilic inflammatory infiltrate (i), necrosis (n), and angiogenesis (an) are seen. **(E)** Rabbit of G4. Collagen fibers (c) and angiogenesis (an) are visualized, characterizing the granulation tissue. **(F)** Rabbit of G4. Note granulation tissue (g), necrosis (n), heterophilic inflammatory infiltrate (i) and angiogenesis (an).

G1 animals presented normal skin, with no histological changes in the skin layers. All envenomed animals presented skin lesions. G2 presented multifocal to coalescent areas of necrosis at the epidermis, superficial, and deep dermis, extending to the hypodermis, with tissue loss and accentuated heterophilic inflammatory infiltrate. Some animals also presented evidence of dystrophic mineralization. G3 animals had intense heterophilic inflammatory infiltrate, necrosis, and angiogenesis, while G4 rabbits had necrosis, heterophilic inflammatory infiltrate, granulation tissue, and angiogenesis. In this study, the intravenous administration of secretome, demonstrated by G4, was more promising due to the absence of dermonecrotic wounds in three out of the four animals tested. All animals in G4 showed the formation of granulation tissue, composed of small and tortuous vessels perpendicular to the epidermis (angiogenesis), and the proliferation of fibroblasts with collagen formation parallel to the epidermal surface (fibroplasia). Among the studied groups, G4 was the only one in which all animals presented granulation tissue, angiogenesis, and fibroplasia in histopathology (Figure 3).

## 4. Discussion

This study aimed to evaluate the protective effect of mesenchymal stromal cell-derived products (secretome) on dermonecrosis induced by *Loxosceles intermedia* spider venom in rabbits. Skin damage due to loxoscelism is very significant, due to the extent of the wound and slow healing process. Antivenoms, although important, have limited action towards dermonecrosis [3]. There is a crucial and urgent need for newer, more efficacious treatments to enhance the healing process and achieve optimal outcomes morphologically and functionally. 

In this study, we determined that experimental *L. intermedia* envenoming causes important lesions in rabbits’ skin, with clinical presentation after 72 hours and reduced progression between the ninth and fifteenth days. The treatment proposed, secretome, by intravenous route, promoted angiogenesis, at the histological level. The secretome is a complex mixture of proteins, including growth factors, cytokines, and vesicular fractions, that have active pharmaceutical potential [30]. In a pilot study, Sanchez-Castro and collaborators showed preliminary results that secretome reduced myotoxicity induced by *Bothrops atrox*, favoring a more successful regenerative response [9]. Furthermore, secretome derivatives, such as conditioned media or exosomes, may present considerable advantages over cells for manufacturing, storage, handling, product shelf life, and their potential as a ready-to-go biologic product [21].

Our results demonstrated that intradermal application of secretome alone did not cause hemorrhage or necrosis, however persistent edema occurred like Sanchez-Castro [9]. At histological evaluation, 15 days after application, no alterations were found. These findings represent an important step towards understanding secretome pharmaceutical potential and reaffirm that the local side effects are minimal. 

Considering the secretome potential, we used two different application methodologies, intradermic and intravenous, to test the therapeutic effect of loxoscelism. Despite the rapid appearance of the hemorrhagic halo (Figure 1) and edema, intravenous administration of the secretome (G4) contributed to the non-development of necrosis in three of the four animals tested. 

Histopathological findings after staining with hematoxylin-eosin (HE) in animals from G2, injected with *L intermedia* venom and treated with only id saline, showed characteristics of focally extensive necrotizing and heterophilic dermatitis, and panniculitis with accentuated multifocal hemorrhage (Figure 3 B ), which are expected in lesions associated with *Loxosceles* [8, 31, 32]. Multifocal thrombosis, moderate edema, granulation tissue formation, and dystrophic mineralization were visualized, which were also reported in other research that also studied rabbits after manual injection of *Loxosceles intermedia* spider venom [31, 33].

The main cause of dermonecrotic lesions observed in loxoscelism is intense heterophilic tissue invasion [34, 35]. This can be seen in all groups in this study, except for the G1. In G3, numerous vessels markedly dilated with a lumen filled with red blood cells (considerable hyperemia), separation of collagen fibers with accumulation of eosinophilic amorphous material (edema), and multifocal to coalescent areas with numerous extravasated red blood cells (marked hemorrhage) were also observed. The present results are similar to those of Elston *et al*. [31] and Ospedal *et al*. [33].

The histopathological findings related to group G4 were similar to groups G2 and G3. Focally extensive necrotizing and heterophilic panniculitis and dermatitis, accentuated with marked multifocal hemorrhage, multifocal thrombosis, dystrophic mineralization, moderate edema, and granulation tissue formation were observed. In this group, only one animal presented necrosis in the epidermis, while all other animals had necrosis from the deep dermis. G4 animals presented fewer macroscopically evident lesions, and only one developed a dermonecrotic scar, however, intense heterophilic infiltrations were observed in all animals. Wound healing is a highly sequential process of skin barrier function restoration and consists of temporally overlapping and interdependent phases, including hemostasis, inflammation, proliferation, and tissue remodeling. During these phases, there are dynamic interactions between numerous different types of skin cells and immune cells that function at specific stages to reshape the wound healing process [36, 37]. 

Histological analysis also revealed that secretome in both groups promoted angiogenesis, but intravenous administration also caused a proliferation of fibroblasts. Fibroplasia is considered the main milestone of the healing process, being an event marked by the migration of fibroblasts in the wound due to the release of chemical mediators produced mainly by macrophages, such as Transforming Growth Factor-alpha (TGF-α). These cell groups are the main components of the granulation tissue and their fundamental function is to produce collagen that will form the connective tissue, replacing the temporary extracellular matrix with a stronger and more elastic tissue, increasing tissue resistance [38]. It was stated that neovascularization enables the formation of essential conditions for metabolically active processes to evolve fully [39]. The injured tissue is then filled by hyperplastic tissue, the granulation tissue, which is mainly composed of macrophages, fibroblasts, and neoformed vessels. They are supported by a loose matrix of fibronectin, hyaluronic acid, and collagen type I and II [40, 41].

These observations in animals treated with MSCs can be explained by the immunomodulatory effect present in MSCs, which attenuates inflammation and reprograms the local immune system, enabling tissue repair and inhibiting the formation of exuberant fibrotic tissue [42, 43]. A correlation with MSCs is performed, as the secretome was classified by Gnecchi and collaborators [44], as trophic molecules released by MSCs that mediate their therapeutic effects and tissue repair [9, 45, 46].

The underlying mechanism may also be associated with the fact that adMSCs cultured in a xeno-free and human component free culture media (XANADU medium) produce a secretome containing proteins such as, Tissue Inhibitor of Metalloproteinase-1 (TIMP-1) and Tissue Inhibitor of Metalloproteinase-2 (TIMP-2), natural inhibitors of matrix metalloproteinases (MMPs) with an important role in the establishment of the right balance to promote tissue remodeling; Angiogenin, a potent stimulator of angiogenesis; CC-chemokine Regulated upon Activation Normal T cell Expressed and Secreted (RANTES/CCL5), Eotaxin, Monocyte Chemoattractant Protein 1 (MCP1), Epithelial Cell-derived Neutrophil-activating Peptide 78 (ENA78), Growth Regulated Oncogene-α (Gro-α), Interleukin 6 (IL6), Interleukin 8 (IL8), and Glutamate Carboxypeptidase II (GCP2) which are chemokines that regulate the immune response, act on leukocytes through selective receptors in maturation, trafficking, and homing of these cells, among others [9, 25]. All these results are consistent with several studies that describe that secretome composition includes proteins related to wound healing and to the angiogenesis stimulation at the wound site, in addition to growth factors, cytokines, and a vesicular fraction, composed of microvesicles and exosomes [21, 46, 47]. These molecules are involved in the transfer of proteins and genetic material, such as micro RNAs, to other cells, with promising therapeutic effects [9, 45, 46, 48]. 

It should be noted that secretome properties can be sensitive to different factors, such as: (a) administration route (subcutaneous, intravenous, or topical application); (b) etiology of lesion formation; (c) basal medium used to collect the secretome and; (d) concentration used [49]. The presented results indicate the need to reassess the parameters used, test other doses and periods of treatment, and determine the administration routes for the treatment. 

Although the secretome could not completely prevent the development of macro and microscopical effects caused by *L. intermedia* venom, it seems to have promoted a microenvironment more favorable to wound healing. A longer observation time would have been important to verify the healing process to its completion, comparing the time needed to achieve this and the composition of the scar tissue formed. Moreover, the impossibility of having a larger number of animals makes the biological variation of the individuals a limitation of this study. The small sample size does not allow us to monitor the animals’ histology throughout the wound healing period, in order to continue with a minimal sample number for performing the statistical evaluation, as the animals were euthanized and histology was made only in the last time point. It is suggested that MSCs-derived products can be important for treating dermonecrotic lesions caused by brown spider bites. Especially, as the secretome is freeze-dried, the possibility of clinical use is great, as it is easily stored.

### 5. Conclusions

The results presented in this work suggest that the therapeutic protocol established with intravenous secretome, obtained from mesenchymal stromal cells derived from human adipose tissue, cultivated in a patented xeno-free and human component-free culture media (XANADU media), to treat *Loxosceles intermedia* venom-induced dermonecrosis provided a reduction in acute inflammation due to the non-formation of necrotic scar in three of the four animals. Treatment with secretome also promoted fibroblast activation, early development of connective tissue, neovascularization, and tissue re-epithelialization, providing an effective alternative in relation to the healing process. This study is a pioneer in the use of MSCs secretome as a treatment for dermonecrotic lesions of cutaneous loxoscelism. Further studies, especially regarding concentrations and time points, should be carried out for the treatment of wounds caused by *Loxosceles* venom.

### Abbreviations

adMSCs: Adipose-derived mesenchymal stem/stromal cells; ENA78: Epithelial cell-derived neutrophil-activating peptide 78; GCP2: Glutamate carboxypeptidase II; GRO-α: Growth regulated oncogene-α; G1: Group 1; G2: Group 2; G3: Group 3; IL6: Interleukin 6; IL8: Interleukin 8; ID: Intradermic; IV: Intravenous; MCP1: Monocyte chemoattractant protein 1; MMPs: Matrix metalloproteinases; MSCs: Mesenchymal stem/stromal cells; NaCl: Sodium chloride solution - saline solution; PBS: Phosphate buffered-saline; RANTES/CCL5: CC-chemokine Regulated upon Activation, Normal T cell Expressed and Secreted; TGF-α: Growth factor-alpha; TIMP-1: Tissue inhibitor of metalloproteinase-1; TIMP-2: Tissue inhibitor of metalloproteinase-2; 

## Availability of data and materials 

 The datasets generated and/or analyzed during the current study are available from the corresponding author upon reasonable request.
